# Extracellular vesicles of *Limosilactobacillus fermentum* SLAM216 ameliorate skin symptoms of atopic dermatitis by regulating gut microbiome on serotonin metabolism

**DOI:** 10.1080/19490976.2025.2474256

**Published:** 2025-03-03

**Authors:** Hyejin Choi, Min-Jin Kwak, Youbin Choi, An Na Kang, Daye Mun, Ju Young Eor, Mi Ri Park, Sangnam Oh, Younghoon Kim

**Affiliations:** a Department of Agricultural Biotechnology and Research Institute of Agriculture and Life Science, Seoul National University, Seoul, Korea; b Food Functionality Research Division, Korea Food Research Institute, Wanju-gun, Jeollabuk-do, Korea; c Department of Functional Food and Biotechnology, Jeonju University, Jeonju, Korea

**Keywords:** Atopic dermatitis, gut-skin axis, extracellular vesicle, gut microbiome, multi-omics analysis

## Abstract

Atopic dermatitis (AD) is a globally prevalent chronic inflammatory skin disorder. Its pathogenesis remains incompletely understood, resulting in considerable therapeutic challenges. Recent studies have highlighted the significance of the interaction between AD and gut microbiome. In this study, we investigated the effects of probiotic-derived extracellular vesicles on AD. Initially, we isolated and characterized extracellular vesicles from *Limosilactobacillus fermentum* SLAM 216 (LF216EV) and characterized their composition through multi-omics analysis. Gene ontology (GO) and pathway analysis classified LF216EV proteins into biological processes, molecular functions, and cellular components. Importantly, specific abundance in linoleic, oleic, palmitic, sebacic, and stearic acids indicating upregulated fatty acid metabolism were observed by metabolomic analysis. Furthermore, featured lipid profiling including AcylGlcADG and ceramide were observed in LF216EV. Importantly, in an atopic dermatitis-like cell model induced by TNFα/IFNγ, LF216EV significantly modulated the expression of immune regulatory genes (TSLP, TNFα, IL-6, IL-1β, and MDC), indicating its potential functionality in atopic dermatitis. LF216EV alleviated AD-like phenotypes, such as redness, scaling/dryness, and excoriation, induced by DNCB. Histopathological analysis revealed that LF216EV decreased epidermal thickness and mast cell infiltration in the dermis. Furthermore, LF216EV administration reduced mouse scratching and depression-related behaviors, with a faster onset than the classical treatment with dexamethasone. In the quantitative real-time polymerase chain reaction (qRT-PCR) analysis, we observed a significant increase in the expression levels of *htrb2c*, *sert*, and *tph-1*, genes associated with serotonin, in the skin and gut of the LF216EV-treated group, along with a significant increase in the total serum serotonin levels. Gut microbiome analysis of the LF216EV-treated group revealed an altered gut microbiota profile. Correlation analysis revealed that the genera *Limosilactobacillus* and *Desulfovibrio* were associated with differences in the intestinal metabolites, including serotonin. Our findings demonstrate that LF216EV mitigates AD-like symptoms by promoting serotonin synthesis through the modulation of gut microbiota and metabolome composition.

## Introduction

Atopic dermatitis (AD) is characterized by chronic skin inflammation and can affect both pediatric and adult populations.^1^ The pathogenesis of AD remains elusive; however, various studies have implicated a potential interplay of diverse factors, including genetic predisposition, dysregulated innate and adaptive immune responses, environmental influences, and compromised epidermal epithelial function.^2–5^ The incidence of AD during childhood can increase the risk of subsequent allergic conditions, including asthma, rhinitis, and food allergies.^6^ Moreover, scratching in response to AD can disrupt the skin barrier, and the associated itching can impair sleep quality, potentially hindering growth in children during critical developmental stages.^7^ Topical corticosteroids and systemic immunosuppressants are the primary treatments for AD. However, long-term clinical data of topical corticosteroids for pediatric use are lacking, and systemic medications can result in notable side effects and pose a risk of relapse when the treatment is discontinued.^8^ Owing to the limitations of these treatments, various studies are ongoing to develop innovative therapies with reduced side effects for the management of AD in both pediatric and adult populations.

Recent studies have established a correlation between the gut microbiome and AD pathogenesis, and Reddle et al. demonstrated notable differences in the overall structure of the gut microbiome between individuals with and without allergic conditions.^4^ Furthermore, alterations in the gut microbiome can influence a range of factors, including metabolism, immunity, development, and others.^9^ Accordingly, recent studies have explored diverse therapeutic strategies aimed at modulating the gut microbiota, including the use of probiotics and metabolites, to mitigate AD.^10,11^ Therefore, altering the composition of gut microbiome in patients with AD has emerged as a promising therapeutic approach for its treatment.^12^

Extracellular vesicles (EVs) are nanosized vesicles that carry bioactive substances, such as proteins, lipids, and nucleic acids, which are potentially involved in the pathophysiological mechanisms of the host. In particular, EVs are enveloped by lipid bilayers, and they can traverse blood vessels, mucus layers, and various tissues, or migrate directly to these sites. Thus, they can facilitate the swift delivery of bioactive substances capable of eliciting potent cellular responses.^13^ EVs are also recognized as safe and effective means of delivering the beneficial effects of probiotics, while avoiding the potential side effects observed in diverse animal models.^14,15^ Recent therapeutic applications of probiotics have highlighted probiotic-derived EVs as novel substances.^16,17^ These EVs offer faster effectiveness and fewer side effects in the therapeutic context, facilitating new modes of communication between the host and microbes.^18^ Recent studies have shown that probiotic-derived EVs alleviate gastrointestinal disorders by modulating specific pathways.^14,19^ Furthermore, their potential use as therapeutic agents to treat stress-induced behaviors has been reported, highlighting their promise as functional alternatives to next-generation probiotics.^20^ In this study, we isolated and characterized EVs (LF216EV) from a potential probiotic strain, namely, *Limosilactobacillus fermentum* SLAM216, validated the function of LF216EV *in vitro* using *a Caenorhabditis elegans* model and human epidermal keratinocyte (HaCaT) cell line, and evaluated its efficacy in alleviating AD symptoms using a 2,4-dinitrochlorobenzene (DNCB)-induced AD mouse model.

## Materials and methods

### Bacteria strains and culture conditions

*L. fermentum* SLAM216, isolated from the feces of Korean infants, was cultured in De Man, Rogosa, and Sharpe broth (MRS; BD Bioscience, Franklin Lakes, NJ, USA) for 24 h at 37°C, following protocols described in previous studies.^21,22^ *Escherichia coli* OP50, the standard feed for *C. elegans*, was grown in Luria-Bertani (LB; BD Biosciences, NJ, USA) broth with shaking at 225 rpm for 24 h at 37°C.^23^ For the killing assay, the pathogenic bacteria *Staphylococcus aureus* Newman and *E. coli* O157:H7 were cultured in Tryptic Soy broth (TSB; BD Biosciences, Sparks, MD, USA) for 24 h at 37°C. All bacterial strains were sub-cultured twice before use. Cultured bacteria were centrifuged at 4,000 rpm for 10 min to collect the pellet, washed twice with phosphate-buffered saline (PBS) and M9 buffer, and subsequently centrifuged at 4,000 rpm for 10 min to remove the supernatant. The bacterial suspensions were then adjusted to a concentration of 1.0 × 10^9^ CFU/mL in PBS and M9 buffer prior to use.^24^

### Isolation and concentration of extracellular vesicles using super absorbent polymer (SAP) beads

EV isolation was performed in accordance with the recently updated Minimum Information for Study of Extracellular Vesicles (MISEV 2023) guidelines.^25^ LF216EV was isolated using previously described protocols with modifications to the SAP bead concentration and the polyethylene glycol (PEG) method.^26–29^ LF216 cultures grown in MRS medium for 24 h at 37°C were centrifuged at 4,000 × g for 10 min at 4°C to separate the supernatants.^30^ The separated supernatants were enriched using SAP beads (CAS 9003-06-9; LG Chem, Korea) purchased from Tippi, Korea (www.tpy21c.co.kr). Before use, the beads were consecutively washed thrice with ethanol and dried in an oven at 60°C. SAP beads with a concentration of 10% were then added to the bacterial supernatants and stored at 4°C for 1 h. After separating the SAP beads from the enriched culture, the culture was filtered through a 0.22-µm filter following centrifugation at 6,000 × g for 20 min at 4°C. PEG-6000 (Sigma Aldrich, St. Louis, MO, USA) was added to the filtered culture at a concentration of 15% of the final volume and incubated overnight at 4°C. After centrifugation at 8,000 × g for 30 min at 4°C, the collected pellet was resuspended in PBS and ultracentrifuged at 150,000 × g for 60 min at 4°C for washing step. The final pellet was suspended in PBS, filtered through a 0.22-µm filter, and its concentration was determined by nanoparticle tracking analysis (NTA) using the NanoSight NS300 (Malvern, UK). The isolated EVs were then stored at −80°C until use.

### Scanning electron microscopy (SEM) and transmission electron microscopy (TEM) analysis

SEM and TEM were conducted for the visual analysis of LF216EV. To scan LF216EVs on the bacterial surface, LF216 was first fixed using Karnovsky’s fixative at 4°C, followed by fixation with 1% osmium tetroxide in 0.05 M sodium cacodylate buffer (pH of 7.2).^31^ Subsequently, dehydration was realized using ethanol solutions of increasing concentrations (30, 50, 70, 80, 90, and three rounds of 100%). Hexamethyldisilazane (100%) was used for the initial drying process of the specimen for two rounds of 15 min each, followed by the use of isoamyl acetate hexamethyldisilazane (100%) for the second drying process of the specimen for two rounds of 15 min each. The prepared samples were mounted on stubs and examined by SEM (JSM-5410LV, Tokyo, Japan). For TEM investigations, a previously described method was employed.^32^ First, 5 μL of the LF216EV sample were deposited onto a carbon-coated grid and allowed to settle for 60 s. The samples were then negatively stained with 2% uranyl acetate and observed by TEM (JEM1010, JEL, Japan).

### Multi-omics analysis

#### Proteomics analysis

The protein composition of EVs was analyzed using a previously reported method.^33^ Purified LF216EV pellets and LF216 whole cells were sonicated using a sonication device (KSS-N1800DT, KOREA PROCESS TECHNOLOGY, Seoul, Korea) for 15 cycles, with each cycle comprising 30 s of sonication followed by switching off for 30 s. Before in-solution digestion, the total protein concentration was quantified using the Bradford assay (Bio-Rad, Hercules, CA, USA). Subsequently, 100 μg of extracted EV proteins and bacterial sample were used for the proteomics analysis. Samples were digested further by trypsin (Gibco, NY, USA) at 37°C overnight. The resulting peptides were purified using SepPak C18 cartridges (Waters Corporation, Milford, MA). Protein profiling was conducted using multi-omics high-resolution mass spectrometry (Orbitrap Exploris 240, Thermo Fisher Scientific, USA).^34^

#### Lipidomics analysis

Lipid analysis was conducted as previously described with minor modifications.^13,35^ For lipid extraction from EVs and bacteria, 5 μL of 0.1% sodium dodecyl sulfate (Sigma Aldrich, St. Louis, MO, USA) in PBS was added to 45 μL of each sample in PBS, resulting in a final concentration of 0.01% sodium dodecyl sulfate (SDS) in PBS. The mixture was then incubated for 20 min at 37°C. After sonication, with each cycle comprising 5 s of sonication followed by switching off for 10 s (for a total of 3 min on ice using a probe sonication device), the lysed samples were centrifuged at 13,500 × g for 20 min at 4°C. The samples were vortexed thrice for 30 s each and subsequently incubated for 10 min at room temperature. Following centrifugation for 2 min at 13,800 × g and 4°C, the supernatant was transferred to a new tube. The remaining pellet was dissolved in chloroform/methanol/37% HCl (40:80:1), incubated for 15 min at room temperature, and vortexed thrice for 30 s. Subsequently, 250 and 450 μL of chloroform and 0.1 M HCl, respectively, were added to the sample. The sample was vortexed vigorously for 1 min and centrifuged at 6,500 × g for 2 min at 4°C. The bottom organic phase was collected and pooled, as previously described. Subsequently, all samples were divided in half and dried using a Speed Vac concentrator. They were then dissolved in 100 μL of 80% methanol for lipid analysis.^36^ Lipid analysis was conducted using a nano high-resolution liquid chromatography-tandem mass spectrometry (LC/MS/MS) spectrometer (Q Exactive, Thermo Fisher Scientific, USA).

#### Metabolomics analysis

Metabolomic analyses were conducted according to previously reported methods.^37^ The bacterial, EV, and mouse cecal samples were mixed with ice-cold methanol. After centrifugation at 10,000 rpm for 10 min at 4°C, the supernatant was passed through a polyvinylidene fluoride syringe filter (Whatman, Maidstone, England) with a pore size of 0.2 μm. The filtered supernatant was concentrated to dryness using a vacuum concentrator. For derivatization, the extract was incubated with 30 µL of a 20-mg/mL solution of methoxyamine hydrochloride in pyridine (Sigma, St. Louis, MO, USA) for 90 min at 30°C, followed by the addition of 50 µL of N,O-bis(trimethylsilyl)trifluoroacetamide (Sigma, St. Louis, MO, USA) for 30 min at 60°C. The gas chromatography-mass spectrometry (GC-MS) analysis was conducted using a Thermo Trace 1310 GC system (Waltham, MA, USA) coupled with a Thermo ISQ LT single quadrupole mass spectrometer (Waltham, MA, USA). After analysis, the data were processed with the Thermo Xcalibur software using automatic peak detection, and metabolites were identified using the National Institute of Standards and Technology (NIST) mass spectral search program (version 2.0, Gaithersburg, MD, USA) by matching their mass spectra with retention indices.^38,39^ Further analyses were conducted using Metaboanalyst version 5.0 (http://www.metaboanalyst.ca).

#### Small RNA-sequencing for microRNA (miRNA) analysis

For miRNA analysis of LF216EV, miRNAs were isolated using the miRNeasy Mini Kit (Qiagen, Hilden, Germany) according to the manufacturer’s protocol. The analysis procedure is outlined as follows^29,30^ the amplified library was purified on a 6% Novex Tris/borate/EDTA polyacrylamide gel electrophoresis (TBE-PAGE) gel (Thermo Fisher, MA) to isolate the 138–220-bp fraction, which includes 18–100 and 120 bp of cDNA and adapters, respectively. The resulting cDNA library incorporated the sequences necessary for clustering in an Illumina flow cell. Library validation was conducted through gel purification and the assessment of size, purity, and concentration using an Agilent Bioanalyzer. Libraries were quantified by quantitative polymerase chain reaction (qPCR) in accordance with the qPCR Quantification Protocol Guide (KAPA Library Quantification Kit for Illumina Sequencing Platforms) and further validated using a TapeStation D1000 screen tape (Agilent Technologies, Waldbronn, Germany). Equimolar pooling of libraries was conducted, followed by sequencing on an Illumina HiSeq 2500 system (Illumina, San Diego, CA, USA) to produce base reads. Image decomposition and quality value calculations were conducted using modules within the Illumina platform.

### *Life span and killing assays of* C. elegans

*C. elegans* was maintained on agar plates with nematode growth medium (NGM) supplemented with the *Escherichia coli* OP50 strain at 20°C following standard protocols.^32,40^ The *CF512 fer-15(b26) II;fem-1(hc17)IV* (*fer-15;fem-1* worms) strain was used for the lifespan and killing assays. For the lifespan assay, worms at the L4 young adult stage were exposed to concentrated LF216 and EVs (1.0 × 10^10^ particles) containing OP50, and the number of live and dead worms was recorded every day. For the killing assay, L4 young adults were exposed to enriched LF216, OP50, and EV (1.0 × 10^10^) for 24 h and subsequently transferred to *E. coli* O157:H7 EDL933 and *Staphylococcus aureus* Newman, and live worms were counted daily. The body length and width of *C. elegans* were measured using the wild-type strain. Worms at the L4 stage were exposed to OP50 containing concentrated LF216 and EV (1.0 × 10^10^ particles) for 24 h, and worm growth was measured using the WormLab software (MBF Bio-science, Williston, VT).

### HaCaT cell culture and wound healing assay

HaCaT cells were maintained in Dulbecco’s Modified Eagle Medium (DMEM; Gibco-BRL, Grand Island, NY, USA) supplemented with 10% fetal bovine serum (FBS; Gibco-BRL, Grand Island, NY, USA) and 1% penicillin-streptomycin (PS; Gibco-BRL, Grand Island, NY, USA). Cells were incubated in a CO_2_ incubator at 37°C in a humidified atmosphere containing 5% CO_2_.^41^ To induce atopic-like inflammation, HaCaT cells were treated with tumor necrosis factor-alpha (TNF-α) (Proteintech, Hubei, China) at a concentration of 10 ng/mL and interferon-gamma (IFN-γ) (Proteintech, Hubei, China) at a concentration of 10 ng/mL for 24 h.^42^ For the wound healing assay, cells were seeded in a 12-well cell culture plate and grown to 100% confluency.^43,44^ A sterile 200-µL pipette tip was used to create a scratch across the cell monolayer. The scratched cells were rinsed with Dulbecco’s PBS (DPBS) to remove detached cells. Subsequently, LF216 and LF216EV in DMEM media were added to each well. Wound closure was monitored and imaged at 0, 3, 6, and 12 h using a microscope. The cell wound closure area was quantified using the ImageJ software, and the percentage of wound closure was calculated to be wound area relative to the original wound area.^45^

### Animal administration and DNCB treatment

Male 7-week-old BALB/c mice (18–20 g) were purchased from SamtaCo (Osaka, Korea). All animals were maintained in a specific-pathogen free (SPF) room at a temperature and humidity of 22.5 ± 0.5°C and 42.6 ± 1.7%, respectively, with a 12-h light-dark cycle. Food and water were provided ad libitum. All mice were acclimated for a week before the assay. This study was approved by the Animal Care Committee of Seoul National University (SNU-230612-4). The AD-induced animal experiments lasted for 21 days. The back skin of the mice was shaved and allowed to recover for one day. DNCB was then applied to the back skin, and the treatments were orally administered.^46,47^ BALB/c mice were randomly divided into five groups: normal (vehicle-treated), control (DNCB-induced and vehicle-treated), dexamethasone (DNCB + Dex; 1 mg/kg; 0.01 mL/day), LF216 (DNCB +1.0 × 10^9^ CFU/kg; 0.01 mL/day), and LF216EV (DNCB +1.0 × 10^10^ particles/mL/kg; 0.01 mL/day). Dexamethasone, LF216, and LF216EV were diluted in PBS and administered to mice using feeding needles (Zonde). The normal and control groups were treated with PBS. During 1 week of primary sensitization, PBS was applied to the back skin of the normal group, whereas 1% DNCB was applied to the back skin of the AD-treated group (dissolved in acetone:olive oil = 3:1) (Sigma, St. Louis, MO, USA) every 3 days. During the secondary sensitization period, the normal group was orally administered PBS, whereas the control, LF216, LF216EV, and Dex groups were sensitized with 0.5% DNCB every 2 days, followed by 14 days of oral administration of the respective treatments (Figure 5a). Mice were subjected to behavioral tests on days 19 and 20 and sacrificed on day 21. Blood samples were collected from the heart, allowed to clot for 1 h at room temperature, and subsequently centrifuged at 2000 rpm for 10 min at 4°C to separate the serum. Dorsal skin tissue, cecum, and intestinal samples were collected for genetic and histological analyses.

### Evaluation of the severity of dermatitis

The severity of DNCB-induced dermatitis was scored using criteria established in previous studies.^48^ Dermatitis severity was assessed every 2 days based on the presence of erythema/hemorrhage, edema, excoriation/erosion, and dryness in the skin lesions and categorized as follows: 0, no symptoms, 1, mild symptoms; 2, moderate symptoms; and 3, severe symptoms.

### Mouse open field test

The open field test (OFT) was conducted in a box measuring 50 × 50 × 30 cm, with a camera mounted on top to record the activity of mice.^49,50^ Each mouse was acclimatized to the experimental room for 1 h before testing. The OFT was initiated by placing the mouse in the central area of the box and allowing it to move freely for 10 min. The behavior of mice was continuously recorded using a video camera. After the experiment, mice were returned to their original cages and the apparatus was cleaned with 70% ethanol and rinsed with clean water. The behavior of the mice was analyzed using the EthoVision XT software (Noldus Information Technology).

### Mouse tail suspension test

To identify depression-like behaviors in mice, we conducted a tail-suspension test (TST) following a previously described method.^51,52^ For the test, each mouse was suspended from a rod by securing it 1 cm from the tip of its tail with tape and positioning its head approximately 50 cm away from the horizontal plane. The behavior of the mice was recorded using a video camera. The total duration of the experiment was 6 min, during which the cumulative immobility time was recorded. The immobility time was defined as the time when all limbs of the mouse became immobile, except for breathing. The movement of the mouse was measured in seconds to determine the total immobility time.

### Measuring the scratching behavior of mice

To investigate AD-like scratching behavior in mice, we quantified and recorded instances where the mice rubbed their nose, ears, and dorsal skin with their hind paws. Each mouse was then placed in an observation cage. The scratching behavior was recorded for 10 min immediately after DNCB sensitization on day 20. The recordings were analyzed to count the number of instances of scratching.^53,54^

### Histopathological analysis

The back skin tissue samples of mice were fixed in 10% formalin for one day and embedded in paraffin to produce paraffin blocks. The paraffin blocks were sectioned with a thickness of 4 μm and stained with hematoxylin and eosin (H&E) (Sigma, St. Louis, MO, USA). The histopathological features were observed using light microscopy. Additionally, the sectioned slides were stained with toluidine blue (TB; Sigma, St. Louis, MO, USA) to enumerate the number of mast cells infiltrating the skin.^55^

### Total serum enzyme-linked immune sorbent assay (ELISA)

The total serum immunoglobulin E (IgE; ab157718, Abcam, UK), histamine (ab213975, Abcam, UK), and serotonin (ab133053, Abcam, UK) levels in mice were analyzed using ELISA assay kits.^56^ Serum dilution and experimental procedures were conducted according to the manufacturer’s protocol.

### Quantitative real-time polymerase chain reaction (RT-qPCR) analysis

For RT-qPCR analysis, the dorsal skin and intestinal tissues of mice were digested using the TRIZOL reagent (Invitrogen, Carlsbad, CA, USA), and the tissues were further disrupted with a mini bead beater (Biospec, Bartlesville, OK).^57^ The disrupted tissue was then used to isolate RNA using an RNeasy Mini Kit (Qiagen, Hilden, Germany). Subsequently, 50 ng of total RNA was reverse-transcribed into cDNA using the iScript cDNA synthesis kit (Bio-Rad Laboratories, Hercules, CA, USA), diluted to a concentration of 40 ng/μL using nuclease-free water (Gibco), and used for experiments. RT-qPCR was conducted using a Bio-Rad CFX96 Real-time PCR system (Bio-Rad Laboratories, Hercules, CA, USA) with SsoAdvanced Universal SYBR Green Supermix (Bio-Rad Laboratories) and specific primers for the target genes.^58^ Gene expression in each sample was normalized using glyceraldehyde 3-phosphate dehydrogenase (GAPDH).^59^ The relative fold change in gene expression was calculated using the 2ˆ(-ΔΔCT) method. The PCR primers used for the qRT-PCR analysis are listed in Table 1.^60–63^

### Metagenome analysis for cecum microbiota

To investigate the changes in the mouse gut microbiota, metagenomic analyses were conducted using cecal samples. DNA was extracted from isolated fecal samples using the DNeasy PowerSoil kit (Qiagen, Hilden, Germany) based on the manufacturer’s instructions.^64,65^ The concentration and quality of DNA were assessed by measuring the absorbances at 230, 260, and 280 nm using a spectrophotometer (SpectraMax ABS Plus, Molecular Devices, San Jose, CA, USA). The primer set used for amplifying the V3-V4 region of the 16S rRNA gene was as follows: forward primer: 5’-TCG TCG GCA GCG TCA GAT GTG TAT AGA CAG GTG CCA GCM GCC GCG GTA A-3’; and reverse primer: 5’-GTC TCG TGG GCT CGG AGA TGT GTA TAA GAG ACA GGG ACT ACH VGG GTW TCT AAT-3’. For metagenomic sequencing, pooled libraries were prepared from 1 ng of each sample using a Nextera Flex kit for NextSeq (Illumina, San Diego, CA, USA). The samples were enzymatically sheared, tagged with adapters, PCR-amplified while adding barcodes, purified using columns or beads, and normalized using Illumina beads or manually. Metagenomic sequencing of the 16S rRNA gene was conducted using an Illumina NextSeq 550 platform. Raw data in the “.fastq” format were subjected to demultiplexing, and quality control was conducted to remove samples with quality scores lower than 30. Denoising involves adapter trimming, quality trimming, and error correction of reads to eliminate chimeric sequences. Subsequently, taxonomic classification was conducted, and mitochondrial and chloroplast sequences were removed. Finally, alignment was conducted and the alpha/beta diversity was analyzed.^66,67^

### Statistical analysis

Each data point in this study was analyzed in triplicates, and the data were expressed as the mean ± standard error (SEM) based on three repeated experiments. Statistical analysis involved one-way analysis of variance (ANOVA) followed by Tukey’s posttest, conducted using GraphPad Prism software 10.0. The Student’s t-test was also employed. The significance levels are denoted as *p < 0.05, **p < 0.01, and ***p < 0.001, indicating significant differences across all replicates.

## Results

### Isolation and characterization of probiotic bacteria-derived extracellular vesicles

**Figure 1.**
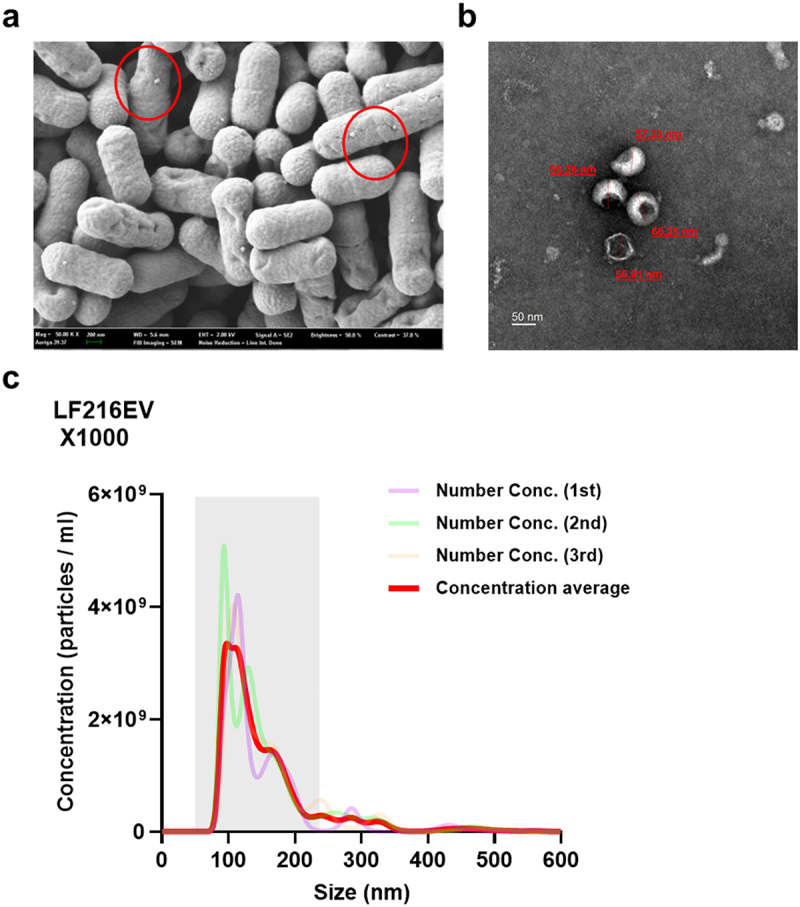
Verification and quantification of LF216EV by SEM, TEM, and NTA analyses. (a) SEM image of LF216 and LF216-derived EV (LF16EV). Scale bar = 200 nm. (b) TEM image of LF216EV particles from LF216. Scale bar = 50 nm. (c) NTA analysis showing the particle range of LF216EV.

The secretion of EVs on the surface of *L. fermentum* JDFM 216 was observed by SEM, and the characteristic morphology of LF216EV was confirmed by TEM (Figure 1(a,b)). Moreover, the size and concentration of LF216EV were assessed using NTA, and the average size of LF216EV was found to be 115.0 nm (Figure 1(c).

### *Comprehensive multi-omics analysis of* L. fermentum *SLAM 216-derived EVs*

**Figure 2.**
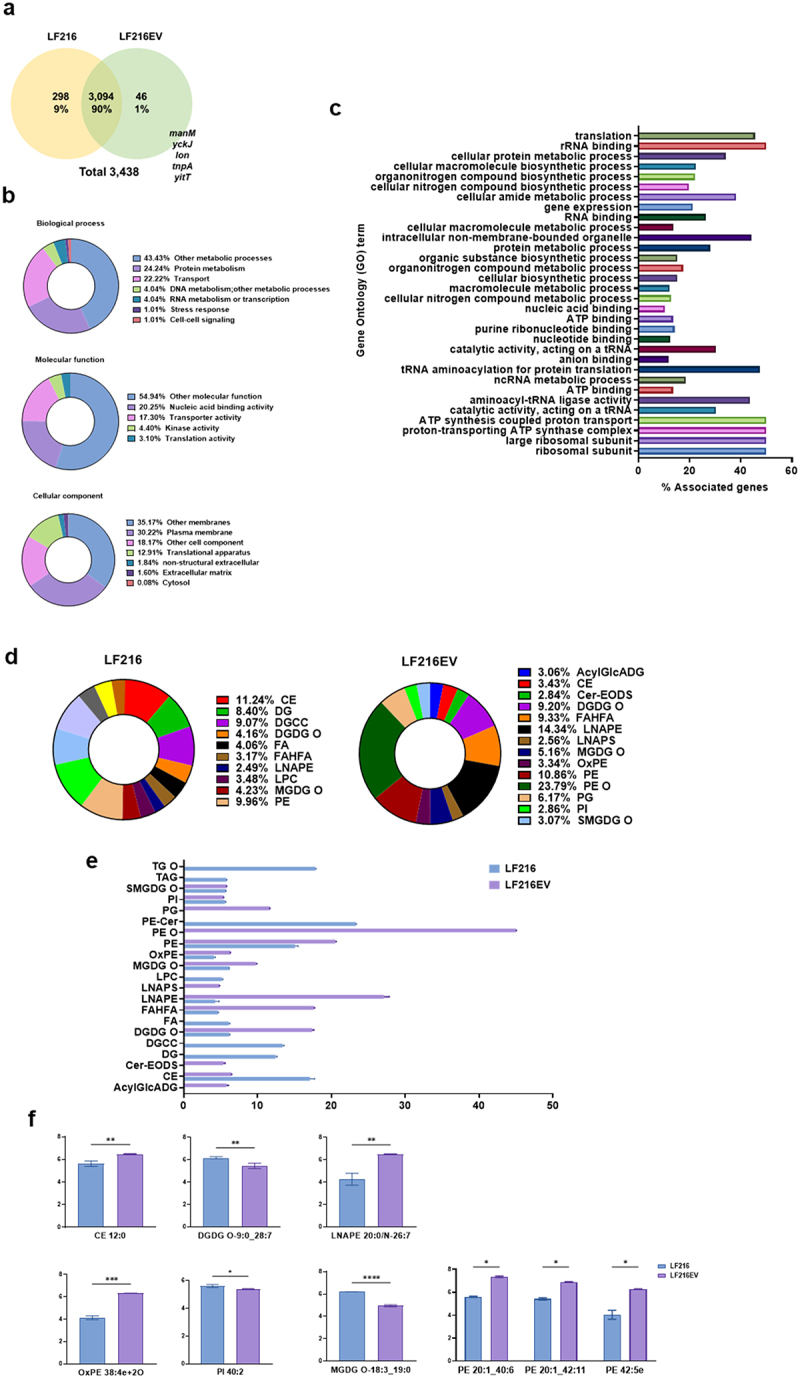
Characterization of LF216EV using proteomics and lipidomics. (a) Proteomic profiling of LF216 and LF216EV. Venn diagram displaying the number of LF216EV proteins identified in LF216. (b) functional classification of identified LF216EV proteins according to the cellular component, molecular function, and biological process. (c) gene ontology (GO) analysis of the identified LF216EV proteins. (d)lipidomic profiling of LF216 and LF216EV. (e) comparison of lipid profiles between LF216 and LF216EV. f relative comparison of the lipid content between LF216 and LF216EV.

The exact components of LF216 and LF216EV were characterized by multi-omics analysis, including transcriptome, proteome, and lipidome analyses. Proteomic analysis revealed 3,392 and 3,140 proteins in LF216 and LF216EV, respectively, and LF216 and LF216EV shared a set of 3,094 proteins (Figure 2(a)). Additionally, 46 proteins were exclusively present in LF216EV, including *manM*, *yckJ*, *Ion*, *tnpA*, and *yitT*. The results from the gene ontology (GO) and pathway analyses revealed protein fragments from LF216EV significantly related to biological processes, molecular function, and cellular component (Figure 2(b)). In particular, LF216EV was related to the plasma membrane and translational apparatus regions in cellular components, metabolic processes, protein metabolism, and transport activities in biological processes. Additionally, LF216EV proteins were significantly associated with nucleic acid-binding activity and transporter activity in molecular functions (Figure 2(c)).

**Figure 3.**
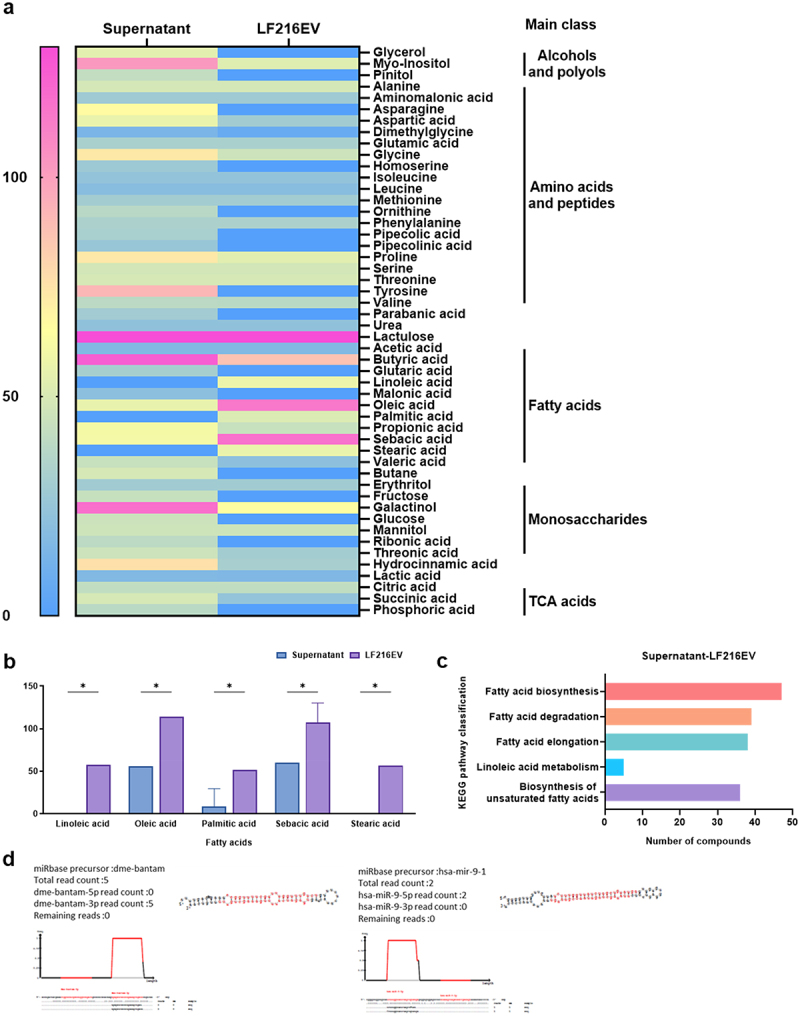
Characterization of LF216EV using metabolomics and miRNA sequencing analysis. (a) overview of the metabolic profiling of LF216 and LF216EV using heatmaps. (b) comparison of the fatty acid classes between LF216 and LF216EV. (c) analysis of the KEGG metabolic pathways related to significantly differential metabolites in LF216EV. (d) miRNA sequencing of LF216EV. Among a total of 771 identified miRNA, Dme-bantam and has-mir-9–1 are the most studied miRNAs among the annotated miRnas.

Through lipid profiling analysis, we identified 27 and 32 lipids in LF216 and LF216EV, respectively; the exact lipid compositions of LF216 and LF216EV are shown in Figure 2(d). AcylGlcADG and ceramides were the major components in the lipidomics of LF216EV but not in that of LF216 (Figure 2(e)). Furthermore, CE, OxPE, LNAPE, and PE were elevated in LF216EV compared with those in LF216, whereas DGDG, PI, and MGDG exhibited lower levels in LF216EV compared with those in LF216 (Figure 2f). Additionally, LF216EV showed significantly increased fatty acid class metabolites compared with LF216, with lower concentrations of monosaccharides, Tricarboxylic acid cycle (TCA) acids, amino acids, and peptides (Figure 3(a)). Linoleic, oleic, palmitic, sebacic, and stearic acids significantly increased in LF216EV, whereas only linoleic and stearic acids were present in LF216EV (Figure 3(b)). Metabolite enrichment analysis also revealed an increase in fatty acid-related metabolic pathways, including fatty and unsaturated fatty acid biosynthesis (Figure 3(c)).

Through miRNA sequencing analysis of LF216EV, we identified 771 matching miRNAs (Figure 3d). Among these, 307 miRNAs were annotated, including bantam linked to neurons and stem cells, mir-263a related to circadian rhythms, and mir-9–1,2,3 associated with human neurons, memory, and neurogenesis.

### *Functional evaluation of LF216EV using an* in-vitro C. elegans *model*

**Figure 4.**
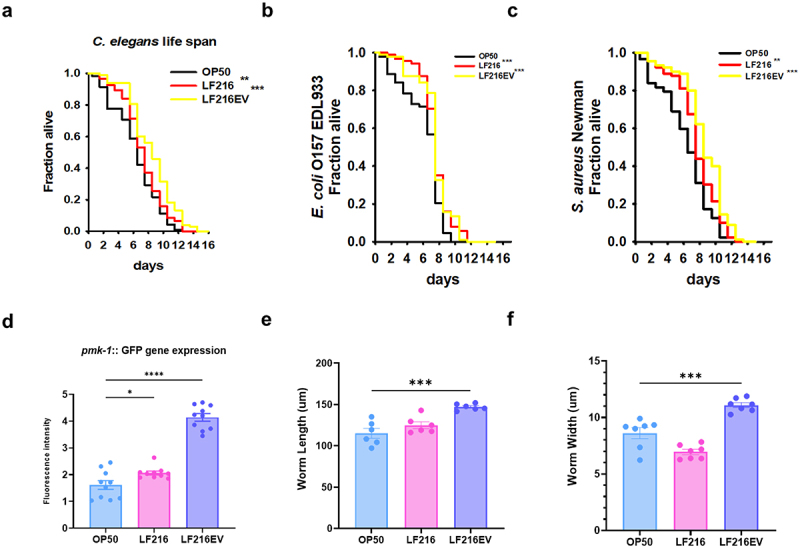
Evaluation of the function of LF216EV in an *in-vivo* model of *C. elegans*. (a) conditioning with LF216 and LF216EV for 24 h prolonged the lifespan of *C. elegans CF512 fer-15(b26)II;fem-1(hc17) IV (fer-15;fem-1)*. (b) stimulating the immunity of *C. elegans* by pre-conditioning with LF216 and LF216EV for 24 h prolonged the lifespan of *C. elegans* exposed to *S. aureus* Newman. (c) stimulating the immunity of *C. elegans* by pre-conditioning with LF216 and LF216EV for 24 h prolonged the lifespan of *C. elegans* exposed to *E. coli* O157:H7 EDL933. d LF216EV induced the expression of the *pmk-1* gene in *C. elegans* AY102 (*p-vha-6::pmk-1*::GFP +*rol-6*(su1006);*pmk-1*::GFP). Data are depicted as the mean values ± SEM of the samples. *p < 0.05, **p < 0.01, and ***p < 0.001 vs. The *E. coli* OP50 control.

The functionality of LF216EV was investigated in an *in-vitro* experiment using *C. elegans*; the extension of worm lifespan by LF216 and its EV was comparable with that by LF216 (*p* = 0.0078 and 0.0005 for LF216 and LF216EV, respectively, compared with the worms fed *E. coli* OP50; Figure 4(a). We investigated the effects of LF216EV on host resistance to foodborne pathogenic bacteria. After 24 h of exposure to LF216EV, pathogen-exposed *C. elegans* specimens exhibited significantly enhanced survival rates compared with those in the control group, and a similar pattern was observed with LF216 (*p* = 0.0001 for *E. coli* O157 EDL 933 and *p* = 0.0000 for *S. aureus* Newman; Figure 4(b,c). Similarly, LF216EV notably upregulated the expression of *pmk-1*, the innate immunity gene in *C. elegans* (*p* = 0.0235 for LF216 and *p* = 0.0000 for LF216EV compared with the worms fed *E. coli* OP50; Figure 4(d). Furthermore, LF216EV promoted worm growth by significantly augmenting both the length and width of *C. elegans* compared with those of worms in the control and LF216 groups (*p* = 0.0002 for length and *p* = 0.0004 for width compared with the worms fed *E. coli* OP50; Figures 4e,f).

### Preventive effects of LF216EV on atopic dermatitis in keratinocytes

**Figure 5.**
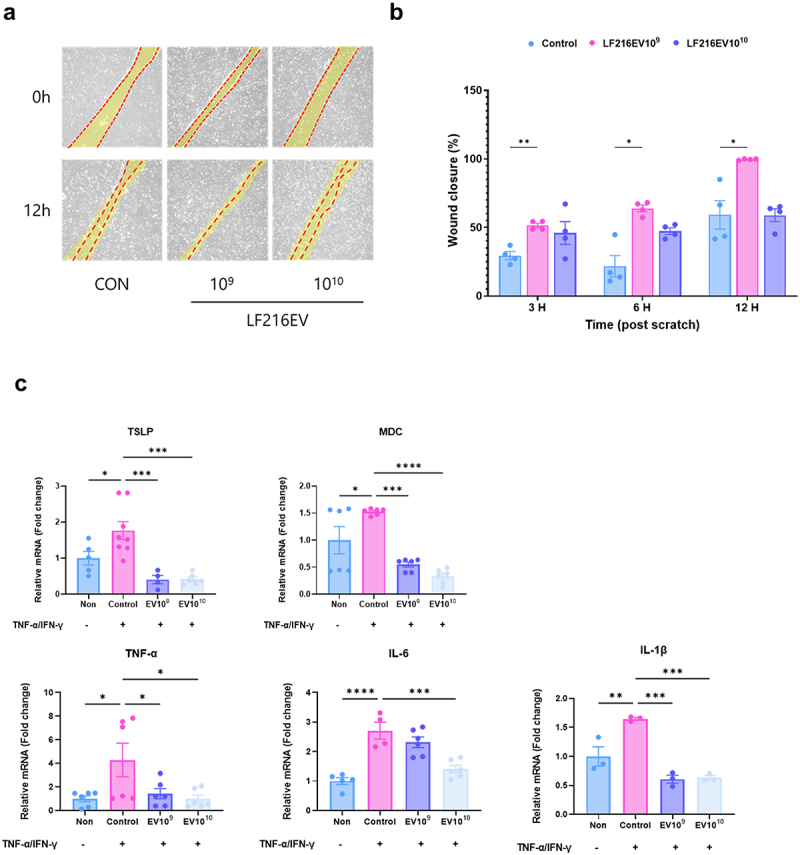
Confirming the functionality of LF216EV in ad-like cell models. (a, b) wound-healing effects of LF216EV in HaCaT cells. LF216EV significantly promoted wound healing of cells after 3 h of treatment. (c) effect of LF216EV on the expression of cytokines by HaCaT cells. HaCaT cells were pre-treated with LF216EV (10^9^, 10^10^ particles/mL) and subsequently treated with TNF-α/IFN-γ for 24 h. TSLP, MDC, TNF-α, IL-6 and IL-1β mRNA expression of HaCaT cells, as detected by qRT-PCR. The mean of three independent experiments is represented by each bar. Data are depicted as mean values ± SEM of the samples. *p < 0.05, **p < 0.01, and ***p < 0.001 vs. The TNF-α/IFN-γ control.

To investigate the effects of LF216EV on keratinocyte repair, a wound healing assay was conducted using the HaCaT cell line. The 10^9^ particles of LF216EV treatment group exhibited a significantly enclosed wounded part of cells 3 h after scratching compared with that of the control group, and complete wound closure was observed after 12 h (*p* = 0.0013, 0.30360, and 0.0127 for 3, 6, and 12 h, respectively, compared with the control; (Figures 5a,b). Furthermore, qRT-PCR analysis related to TNFα/IFNγ-induced atopic-like inflammation revealed that LF216EV significantly downregulated immunoregulatory cytokine, thymic stromal lymphopoietin (TSLP) (*p* = 0.0004 for 10^9^ and *p* = 0.0001 for 10^10^), TNF-α (*p* = 0.0408 for 10^9^ and *p* = 0.0175 for 10^10^), interleukin 6 (IL-6) (*p* = 0.2996 for 10^9^ and *p* = 0.0003 for 10^10^), interleukin-1 beta (IL-1β) (*p* = 0.0270 for 10^9^ and *p* = 0.0325 for 10^10^), and macrophage-derived chemokine (MDC) (*p* = 0.0005 for 10^9^ and *p* = 0.0001 for 10^10^) genes (Figure 5c).

### Effect of LF216EV on the skin lesion of DNCB-induced atopic dermatitis in BALB/c mice

**Figure 6.**
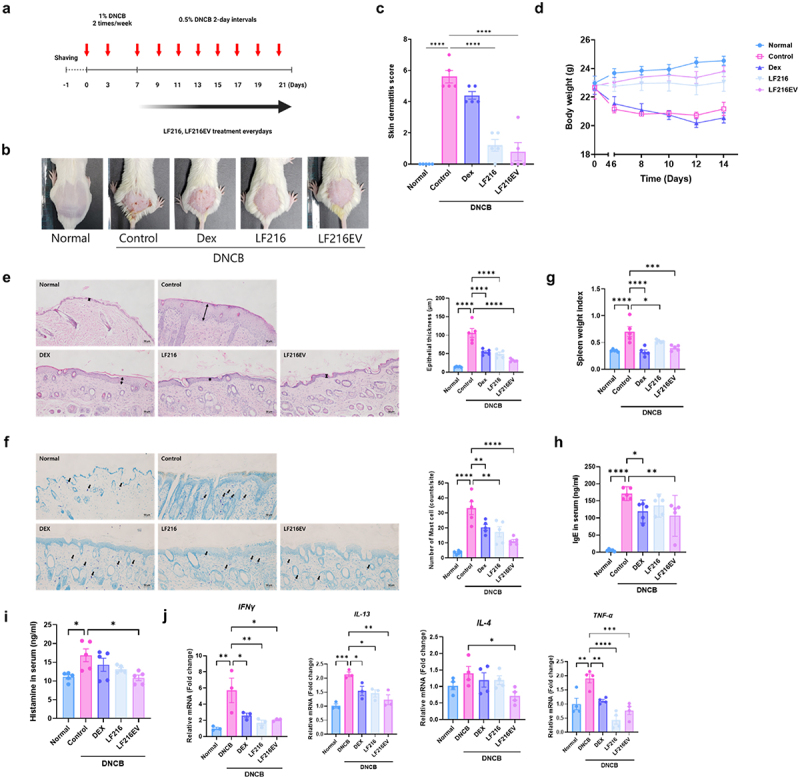
LF216EV attenuates ad-like inflammation in a dncb-induced mouse model. (a) schematic diagram of the experiment. (c) Representative images of the dncb-treated skin area. (c) clinical score (redness, scaling/dryness, and excoriation) of each treatment group. (d) body weight of each treatment group. (e) H&E staining of the dorsal skin lesions from groups of mice and measurement of epidermal thickness. The black arrows indicate epidermal thickness. (f) TB staining and counting of mast cells of the dorsal skin lesions in a group of mice. Black arrows indicate mast cells infiltrating the skin. Scale bar = 50 µm. (g) measurement of spleen weight of each group. (h), i measurement of the total serum concentrations of IgE and histamine. j mRNA expression levels of inflammation-related genes (*IFNγ, IL-13, IL-4*, and *TNF-α*) using qRT-PCR. Data are depicted as mean values ± SEM of the samples. *p < 0.05, **p < 0.01, and ***p < 0.001 vs. The DNCB control.

We conducted an *in-vivo* experiment using a DNCB-induced AD mouse model to investigate the therapeutic effects of LF216 and LF216EV. The experimental scheme is shown in Figure 6a, and representative images of atopic-like clinical symptoms, such as redness, bleeding, scratching, inflammation, and dryness, on the back skin of DNCB-treated mice are shown in Figure 6b. These atopic-like symptoms were significantly reduced by the ingestion of LF216 and LF216EV compared with those in the Dex and control groups (*p* = 0.0001 for LF216 and *p* = 0.0001 for LF216EV compared with the control group; *p* = 0.0001 for LF216 and *p* = 0.0001 for LF216EV compared with the Dex group) (Figure 6c). Moreover, administration of LF216 and LF216EV restored the weight loss induced by AD in mice to levels comparable with those in the normal group (Figure 6d).

To assess the impact of LF216EV on epidermal hyperplasia and mast cell infiltration, back skin tissues of mice were stained and observed using H&E and TB staining. Representative pictures of H&E staining are shown in Figure 6e; the epidermal thickness was significantly lower in the LF216- and LF216EV-treated groups than in the control group by 2.05- and 3.18-fold, respectively (*p* = 0.0001 for LF216 and *p* = 0.0001 for LF216EV compared with the control group). Additionally, representative images stained with TB are shown in Figure 6f, and the DNCB-treated group showed a 7.06-fold increase in the number of infiltrating mast cells compared with the normal group. In contrast, the Dex group showed a reduction in mast cells by 1.24-fold compared with the control group, and LF216- and LF216EV-treated groups exhibited 1.42- and 2.17-fold reductions in in mast cells compared with the control group, respectively (*p* = 0.0001 for LF216 and *p* = 0.0001 for LF216EV compared with the control group).

We also conducted qRT-PCR analysis of skin tissues to elucidate the anti-inflammatory mechanisms of LF216 and LF216EV on AD. In the DNCB-induced AD group, the expressions of IL-13 (*p* = 0.0001), IL-4 (*p* = 0.7082), IFN-γ (*p* = 0.0070), and TNF-α (*p* = 0.0087) significantly increased compared with those in the normal group, and were significantly reduced by the administration of LF216EV compared with those in the control group (*p* = 0.0024 for IL-13, *p* = 00275 for IL-4, *p* = 0.0368 for IFN-γ, and *p* = 0.0007 for TNF-α). Moreover, the expression of pro-filaggrin (FLG) was significantly reduced compared with that in the normal group (*p* = 0.0136). Consistent with previous findings, LF216EV significantly restored the expression of the pro-FLG gene, which decreased by DNCB treatment, to levels comparable with those observed in the normal group (*p* = 0.0406 compared with the control).

To confirm the lowering of systemic inflammation by LF216 and LF216EV administration, the spleen weight and concentration of inflammatory markers in the serum were measured (Figure 6g-i). Consistent with the results of qRT-PCR analysis, LF216EV treatment significantly reduced spleen weight (*p* = 0.0001), with a significant decrease in IgE (*p* = 0.0012) and histamine (*p* = 0.0026) levels compared with those in the control group.

### Administration of LF216EV reduces depressive- and anxiety-like behaviors in AD mice

**Figure 7.**
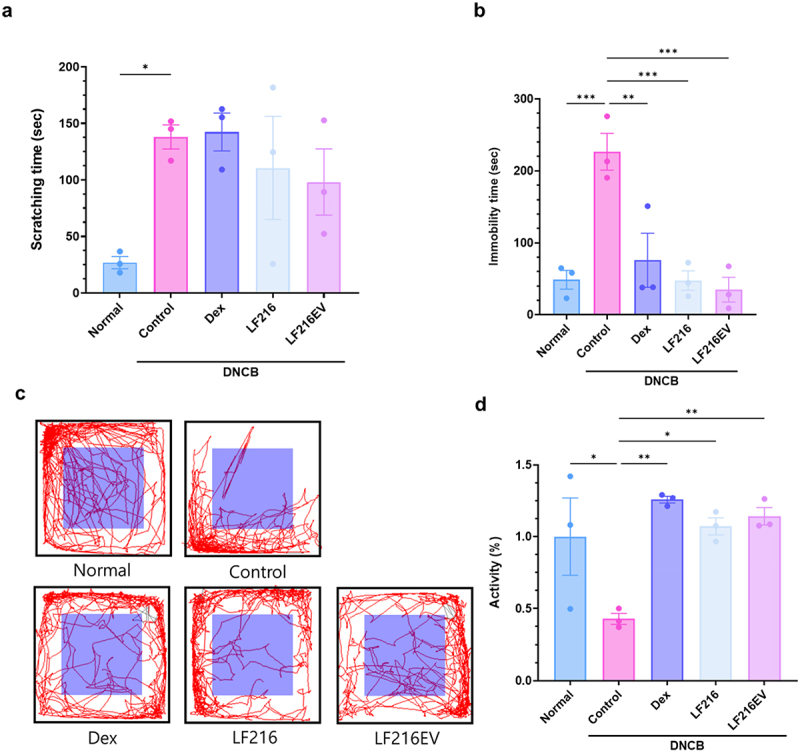
LF216EV alleviates anxiety and depression-related behaviors in a mouse model of dncb-induced ad-like symptoms. (a) the scratch behavior of mice was observed for 10 min following sensitization. (b) LF216EV reduced the immobility time during the TST of mice. (c, d) LF216EV reduced anxiety- and depression-related behaviors in the OFT of mice. d LF216EV induced mouse activity in the OFT. Data are shown as mean values ± SEM of the samples. *p < 0.05, **p < 0.01, and ***p < 0.001 vs. The DNCB control.

Subsequently, we employed scratching, open field, and tail suspension tests to determine whether the mitigating effect of LF216EV on AD helped ameliorate AD-induced aberrant behavior. DNCB-induced AD mice displayed a 5.14-fold increase in scratching time compared with the control group; however, administration of LF216 and LF216EV decreased the scratching times by 1.24- and 1.4-fold, respectively (Figure 7a). Additionally, DNCB-induced AD mice exhibited significantly increased immobility during the tail suspension test (*p* = 0.0006 compared with the normal group); however, the administration of LF216 and LF216EV significantly reduced the immobility time compared with that in the control group (*p* = 0.0006 for LF216 and *p* = 0.0003 for LF216EV; Figure 7b). The results of the open-field test showed a similar pattern, and the control group showed more immobilization time in the corner than the normal group. However, the mice in the LF216 and LF216EV groups moved more toward the center than those in the control group (*p* = 0.0137 for LF216 and *p* = 0.0068 for LF216EV; Figure 7c,d).

### LF216EV induces changes in gut microbiota composition in DNCB-induced AD mice

**Figure 8.**
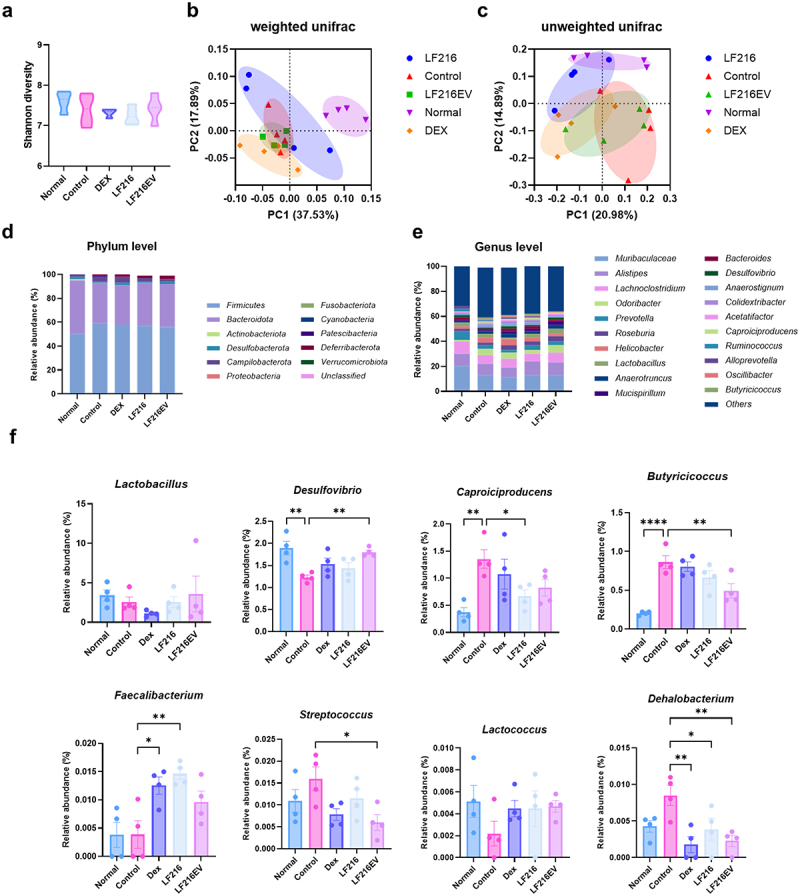
Alteration of gut microbiota in a mouse model of ad-like symptoms by LF216EV. (a) alpha diversity of gut microbiota in each group using the Shannon index. (b, c) the similarity of the bacterial community structure of the gut microbiota using weighted and unweighted UniFrac distances. (d) phylum- and e genus-level gut microbiome compositions of treatment groups. (f) comparison between groups at eight representative genus levels. Data are shown as mean values ± SEM of the samples. *p < 0.05, **p < 0.01, and ***p < 0.001 vs. The DNCB control.

To investigate the relationship between the gut microbial population and physiological and mental symptoms of AD, the microbiome composition of AD-induced mice was determined using 16S rRNA metagenomic analysis. Shannon’s diversity index did not differ between the treatment groups (Figure 8a); however, weighted and unweighted principal coordinate analysis (PCoA) analyses revealed that the plots of gut microbiota between the DNCB and normal treatment groups were distinct, as were those of the Dex-, LF216-, and LF216EV-treated groups (Figure 8b,c).

The gut microbial populations at the phylum and genus levels are shown in Figure 8d,e. At the phylum level, the top phyla differed between the normal control and DNCB-treated groups (DNCB, Dex, LF216, and LF216EV), and the top three dominant phyla in terms of relative abundance for the normal control were *Firmicutes* (50%), *Bacteroidota* (45%), and *Desulfobacterota* (2%), whereas those in the DNCB treatment group were *Firmicutes* (59%), *Bacteroidota* (34%), and *Campilobacterota* (4%). However, in the LF216EV group, the proportions of *Firmicutes* (56%), *Bacteroidetes* (36%), and *Deferribacterota* (3%) were different from those in the other DNCB-treated groups. Similar patterns were observed at the genus level in the control and DNCB-treated groups. DNCB-treated groups showed decreased abundances of *Prevotella* and *Lachnoclostridium* and an increased abundance of *Helicobacter* compared with the normal control. Among the DNCB-treated groups, LF216EV showed a different pattern of *Lachnoclostridium*, *Roseburia*, *Helicobacter*, and *Limosilactobacilli*. The genus-level comparisons indicated that *Desulfovibrio* (*p* = 0.0091), *Butyricicoccus* (*p* = 0.0090), *Streptococcus* (*p* = 0.0183), and *Dehalobacterium* (*p* = 0.0055) were significantly differentiated in the LF216EV group than in the DNCB group (Figure 8f). LF216EV also increased *Limosilactobacillus* (0.71-fold increase compared with that in the DNCB control) and *Lactococcus* (0.46-fold increase compared with that in the DNCB control), which were reduced in the DNCB group (*Limosilactobacillus* and *Lactococcus* decreased by 0.75- and 0.42-fold compared with those in the normal group).

### The metabolome study modulated by LF216EV in DNCB-induced AD mice

**Figure 9.**
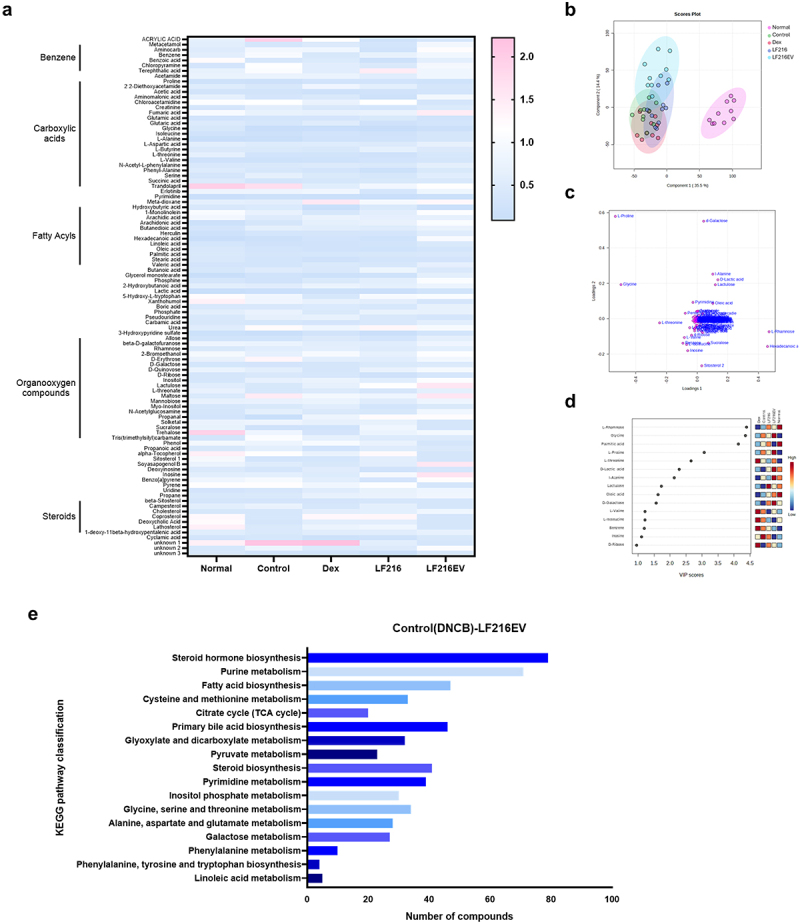
Alteration of gut metabolite changes in a mouse model of ad-like symptoms by LF216EV. (a) effects of LF216EV on the metabolites of gut microbiota in AD mice. Heat map illustrating the levels of gut metabolites. (b) Principal component analysis (PCA) of metabolites in AD mice. (c) differences in metabolites between treatments depicted using PCA loading plots. (d) variable importance in projection (VIP) score of metabolites of AD mice across treatment groups. (e) KEGG pathway classification of the increased metabolites in LF216EV.

In parallel with the shifts observed in the gut microbiome, DNCB treatment induced alterations in the metabolomic profile of the intestinal environment relative to the normal group. A heat map of the metabolite profiles across all experimental groups is shown in Figure 9a. The partial least-squares discriminant analysis (PLS-DA) score and loading plot analysis of metabolite profiles among the experimental treatment groups are shown in Figure 9b,c. DNCB treatment showed a clear cluster separation from the normal groups; however, the clustering of the Dex, LF216, and LF216EV groups shifted from that of the control group. The variable importance in projection (VIP) scores for the top 16 metabolites to distinguish between the groups in the PLS-DA model are shown in Figure 9d. L-rhamnose, glycine, and palmitic acid were identified as the most critical metabolites across all experimental groups, and the administration of LF216EV notably elevated the levels of metabolites, such as glycine, l-proline, lactic acid, and alanine, compared with the those in the other treatment groups. Moreover, we conducted Kyoto Encyclopedia of Genes and Genomes (KEGG) pathway analysis to explore and compare the key metabolic pathways associated with the altered metabolites in the LF216EV-treated group with those in the DNCB control group. In total, we identified 33 pathways, including steroid hormone biosynthesis; phenylalanine, tyrosine, and tryptophan biosynthesis; and glycine, serine, and threonine metabolism (Figure 9e).

### LF216EV alleviates DNCB-induced AD symptoms by regulating serotonin-related genes

**Figure 10.**
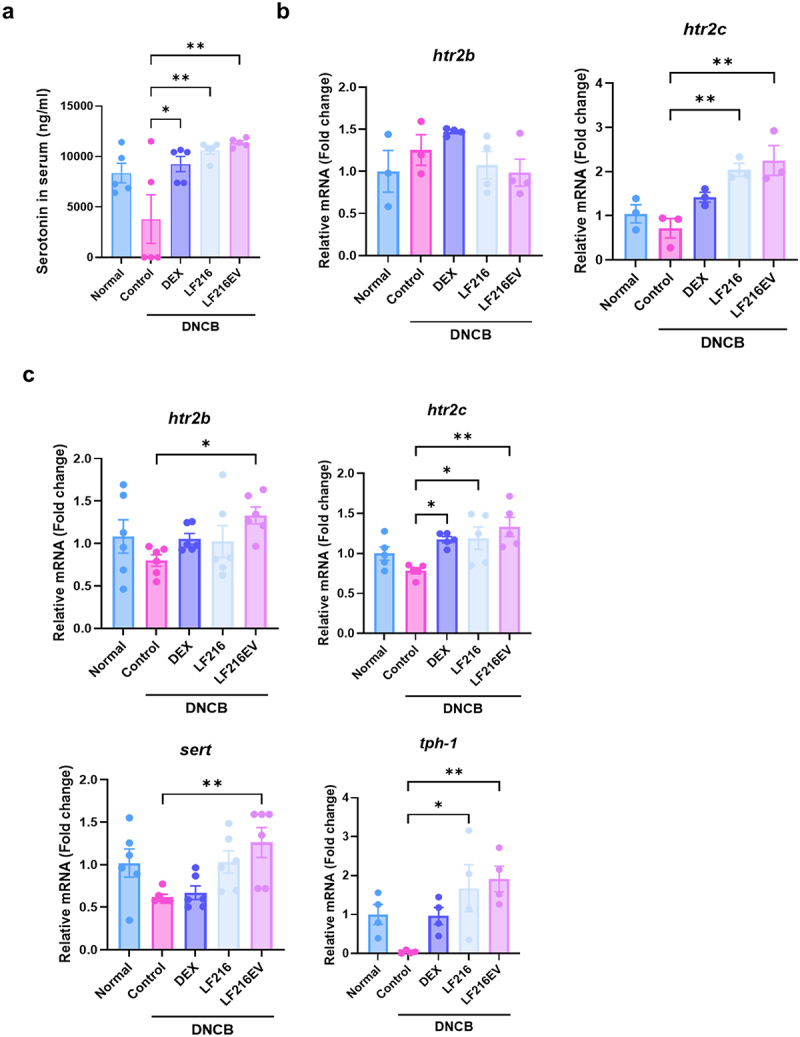
LF216EV modulates the gut and skin serotonin genes to reduce ad-like symptoms in a mouse model. (a) measurement of the total serum concentrations of serotonin. (b) mRNA expression levels of serotonin receptor genes (*htr2b* and *htr2c*) in mice skin using qRT-PCR. (c) mRNA expression levels of serotonin related genes (*htr2b*, *htr2c*, *sert*, and *tph-1*) in mice intestine using qRT-PCR. Data are depicted as mean values ± SEM of the samples. *p < 0.05, **p < 0.01, and ***p < 0.001 vs. The DNCB control.

Analysis of the gut microbiota and gut metabolome in the LF216EV-administration group revealed significant changes in both the bacterial composition and metabolomic profile, particularly those associated with steroid hormone and tryptophan metabolism. Therefore, we hypothesized that the amelioration of DNCB-induced AD-like inflammation and aberrant behavior resulted from alterations in neurotransmitter levels mediated by LF216EV administration and the measured serotonin concentrations in mouse total serum (Figure 10a). Consistent with previous results, LF216EV treatment led to a significant upregulation of serum serotonin concentrations, which were decreased by DNCB treatment (*p* = 0.0005 for LF216EV compared with the control). Additionally, the expression of *htr2b* in mouse skin tissue did not show significant differences among all groups; *htr2c* exhibited a significant increase in both the LF216 and LF216EV groups in the skin (*p* = 0.0039 for LF216 and *p* = 0.0019 for LF216EV) and gut tissues (*p* = 0.0387 for LF216EV; Figure 10b,c). Furthermore, LF216EV administration led to significant upregulation of the serotonin gene, *sert*, and *tph1* in the gut (*p* = 0.0141 for *sert*, *p* = 0.0204 for *tph-1*). Immunohistochemical staining also showed that LF216EV administration significantly increased tph1 protein expression in the gut (*p* = 0.0007) (Supplementary figure S2). Collectively, the administration of LF216EVs can induce symptomatic relief by modulating serotonin-related genes in the intestines of AD mice.

## Discussion

In this study, we explored the efficacy of LF216EV in treating DNCB-induced AD using cells, *C. elegans*, and mouse models. We performed multi-omics analysis to identify the components and functions of LF216EV. The LF216EV is composed of a nanoscale circular lipid bilayer structure, consistent with the morphology observed in previously reported extracellular vesicles (EVs)^68^ (Figure 1). Using multi-omics analysis, we identified unique components of EVs that are different to bacteria (Figures 2 and 3). The 46 proteins present specifically in LF216EV included, among others, TnpA, a target site selection protein, and YitT, which encodes a bacterial membrane protein.^69,70^ Our GO analysis identified associations with plasma membrane and translational apparatus regions in host cellular components, as well as with metabolism and transport. This suggests that EVs could potentially be delivered to the host, increase their binding activity to host nucleic acids, and mediate their function by enhancing the activity of transporters. Lipids are the main component of EVs, forming their lipid bilayers. Lipids play a crucial role in maintaining the structure and stimulating the function of EVs. The LF216EV-specific key lipid component, AcylGlcADG, has been reported in previous studies to have various biological functions, including antiviral, antioxidant, antitumor, and anti-atherosclerotic activities.^71,72^ In addition, ceramide as a major constituent lipid of LF216EV, is formed by the condensation of sphingosine and long-chain fatty acids. It is known to regulate cell death and growth and particularly defense against bacterial-derived infections.^73–75^ Recent studies have also reported that bacterial-derived ceramides are more soluble and permeable to cell membranes compared to animal-derived ceramides. Moreover, various studies are underway to improve skin health by stimulating ceramide synthesis or utilizing the antimicrobial effects of ceramides to improve dermatitis because ceramides are essential components of skin.^73,76,77^ Linoleic acid, a metabolite specific to LF216EV, is increased in intestinal production by lactobacilli and has been reported to inhibit colitis and the growth of foodborne and pathogenic bacteria.^78,79^ The results of this analysis are supported by the killing assay using the *C. elegans* model in this study (Figure 4). The miRNA sequencing results showed patterns similar to the multi-omics results. For instance, bantam is primarily associated with systemic growth and sensory neurons, and has also been reported to exhibit antiviral effects.^80–82^ miR-263a can regulate the host’s circadian rhythm and maintain host osmolality and intestinal stem cell homeostasis by regulating ENaC.^83,84^ It has been reported that miR-9-1,2,3 regulate cell growth through the regulation of FoxP1 and NF-kB, and similar to another miRNAs, they may also regulate neurogenesis, including neurons.^85,86^ Therefore, based on multi-omics analysis, we propose that LF216EV and its components may mediate the modulation and improvement of disease-induced immune responses and mental health in the host.

Thus, we focused on elucidating the mechanisms through which LF216EV mitigates AD-related clinical symptoms, corrects behavioral immune imbalances, and restores skin barrier function. We also examined its effects on scratching behavior, as well as depressive and anxiety-related behaviors. Prior research has indicated that the prolonged use of dexamethasone, a conventional treatment for AD, is linked to adverse effects, such as telangiectasias, skin atrophy, and rebound phenomena.^87,88^ Considering these side effects, probiotics and their metabolites have been proposed as potential alternatives. Nevertheless, the utilization of probiotics has been reported various kinds of side effects, including conflicts with the host’s gut microbiota, inhibition or disruption of crucial eukaryotic host mechanisms, and storage challenges due to maintenance of probiotic functionality in the host.^89–91^ Hence, EVs have been investigated as a means of addressing the constraints of probiotics.^14,15^ EVs offer the potential to deliver probiotic functionality, while also overcoming challenges, such as long-term storage.^92^ Moreover, EVs possess a distinctive targeting ability to specific signaling responses in recipient cells.^93^ In our previous study, oral LF216 ingestion led to enhanced immune response and cognitive behavior by modulation of the gut microbiome in aging mice.^21,22^ Consistent with the effects observed in live bacteria, LF216EV significantly extended the lifespan of *C. elegans* and notably promoted worm growth (Figure 4). It significantly increases the lifespan of worms exposed to dietary pathogen by upregulation of the *pmk-1* gene, which is related to the innate immunity in *C. elegans*.^94^ We also evaluated the impact of LF216EV on host gut health using an intestinal epithelial cell model induced by LPS (Supplementary Figure S1). The results showed that LF216EV significantly upregulated tight junction-related genes (*Claudin1, Muc2, Zo-1, Occludin*) that were deregulated by inflammation. Similarly, immune cytokines (*IL1β, TNFα, IL6*) altered by inflammation were recovered to levels comparable to normal cells. These results indicate that LF216EV can modulate the host’s immune response and promote gut health. Hence, we conducted a cross-check to ascertain whether LF216EV could induce the remission of AD in various cell and animal models.^95^ In line with our hypothesis, LF216EV mitigated the expression of dysregulated immune cytokines in an AD-like cell model and significantly accelerated wound healing (Figure 5). Similarly, LF216EV administration ameliorated the associated clinical symptoms in a DNCB-induced AD mouse model at a faster rate than the other treatments (Figure 6). In particular, AD is characterized by an imbalance in cytokines secreted by Th1 and Th2 cells, which disrupts homeostasis.^96^ In our study, the imbalance induced by DNCB in the LF216EV treatment group was alleviated, suggesting a potential improvement in AD (Figure 6). In AD, the penetration of antigens through defects in the epidermal barrier triggers the increased activity of Th2 cells, characterized by elevated levels of IL-4 and IL-13.^97,98^ This cascade activates the mast cells and eosinophils in the epidermis, ultimately resulting in the development of pruritus. In this study, LF216EV attenuated the expression of Th2 cytokines (IL-13 and IL-4) and significantly decreased the infiltration of mast cells into skin lesions, indicating its potential to alleviate atopic-induced itching. Notably, the total serum histamine levels were reduced, confirming the effectiveness of LF216EV in alleviating pruritus in AD. The total serum IgE level is a typical indicator of the severity of AD.^99^ LF216EV treatment decreased the elevated serum IgE levels in AD, indicating its efficacy as a functional substance capable of promptly alleviating atopic-induced autoimmune abnormalities.

**Figure 11.**
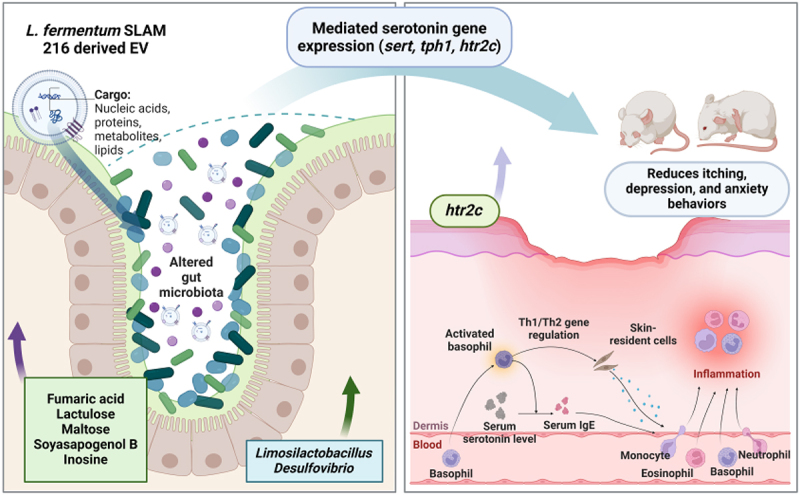
Schematic diagram. The schematic presentation shows the results of LF216EV ameliorating atopic dermatitis by regulating serotonin metabolism via gut microbial modulation. The analysis reveals that LF216EV induced changes in the composition and metabolites of the gut microbiota, particularly the composition of *Limosilactobacillus* and *Desulfovibrio*, and a significant increase in fumaric acid, lactulose, maltose, soyasapogenol B, and inosine. The altered composition of the gut microbiota and metabolome significantly increased the expression of serotonin-related genes *SERT*, *TPH1*, and *HTR2C* in the gut. Similarly, the *HTR2C* gene in skin tissue was upregulated. Notably, the altered gene expression attenuated the phenotype of a mouse model of atopic dermatitis and inflammation-induced scratching, anxiety, and depressive symptoms. Consequently, these effects contribute to the amelioration of atopic dermatitis and the improvement of atopic dermatitis-mediated psychiatric disorders. LF216EV, *Limosilactobacillus fermentum* SLAM 216 derived extracellular vesicles; SERT, serotonin transporter; TPH1, tryptophan hydroxylase 1; HTR2C, 5-hydroxytryptamine receptor 2C.

We also investigated the impact of LF216EV on depressive and anxiety-related abnormalities that often accompany AD (Figure 7). AD is a chronic recurrent disease that typically occurs during infancy or childhood and persists into adulthood. During infancy and childhood, atopy-induced psychiatric disorders and sleep disturbances can have a profound impact on growth and development.^100,101^ In our study, we observed a trend toward increased depressive and anxiety symptoms in DNCB-induced atopic mice. These symptoms were ameliorated by LF216EV treatment. We hypothesized that serotonin, a key neurotransmitter involved in the regulation of mental illness symptoms, might serve as a precursor to this improvement. Consistent with our hypothesis, we observed that the total serum serotonin concentrations were significantly higher in the LF216EV-treated group than in the DNCB-induced AD mice (Figure 10). A significant increase in the serotonin receptor, htr2c, was also detected in the dorsal skin tissues of mice. Similarly, gene expression experiments conducted in the gut tissue, where 95% of serotonin is produced, demonstrated a significant upregulation of serotonin-related genes (*htr2c*, *ser-1*, and *tph-1*).^102^ These results were validated using cellular models (Supplementary Figure S3). In an atopic-like human keratinocyte model, both HTR2B and HTR2C were upregulated in contrast to the mouse model. This finding supports the hypothesis that LF216EV controls the serotonin-mediated alleviation of AD in hosts and regulates depressive- and anxiety-like behaviors (Figure 7). The functionality of this study was mediated through the oral administration of LF216EV. Therefore, we compared the altered profiles of the gut microbiota and gut metabolome in DNCB-induced AD mice (Figures 8 and 9). LF216EV increased the proportions of *Firmicutes*, *Bacteroidota*, and *Deferribacterota*, especially at the phylum level, compared with those in the control group. This is consistent with previous studies reporting a positive association between serotonin levels in brain tissue and the abundances of *Bacteroidota* and *Deferribacterota*.^103,104^ Notably, previous findings have suggested that an increase in both bacteria improved depression-like behavior in chronic stress-induced rats, which is similar to the behavioral experimental results of this study.^103^ LF216EV also increased the abundances of *Limosilactobacillus* and *Lactococcus* at the genus level, suggesting that it may promote the growth of beneficial gut bacteria. In particular, children treated with *Limosilactobacillus fermentum* for 8 weeks exhibited clinical improvement in atopic symptoms, whereas children orally supplemented with *Limosilactobacillus rhamnosus* showed improved total serum IgE levels and AD scores.^105,106^ Adults with an increased *Limosilactobacillus* composition in the gut also exhibited reduced stress-induced depression-like behavior and fewer stress-induced changes in serotonin metabolism.^107^ These changes were also observed in the gut metabolome composition. The LF216EV group exhibited increased levels of fumaric acid, lactulose, maltose, soyasapogenol B, and inosine. Among the altered metabolites, lactulose enhances the production of tryptophan metabolites, which serve as precursors to serotonin in a system that mimics the human gut environment.^108^ Adding maltose to the intestinal perfusate in an *in-vitro* system has been demonstrated to increase the concentration of serotonin in the intestinal perfusate by approximately three-fold.^109^ Soyasapogenol B and inosine have also been reported to act as neuroprecursors. Treatment with inosine has been shown to attenuate the antidepressant effects in mouse models, whereas soyasapogenol B attenuates memory impairment caused by lipopolysaccharide abnormalities in mouse models.^110,111^ This result was further corroborated by the KEGG pathway analysis. LF216EV administration increased the phenylalanine, tyrosine, and tryptophan biosynthesis in DNCB-induced AD mice, suggesting that it can induce serotonin synthesis.^112^ Overall, we propose that LF216EV may serve as a promising anti-AD drug candidate that offers a faster and less toxic alternative to traditional AD treatments, such as dexamethasone and probiotics. We found that the effects of LF216EV were mediated by serotonin signaling. To date, several studies have demonstrated the robust functionality of probiotic-derived EVs, warranting further investigations. Similarly, the serotonin-mediated mechanisms proposed in this study confirm that LF216EV can modulate the immune response and alleviate skin inflammation by altering the gut microbiota of the host. These findings suggest that disease-mediated psychiatric disorders can be ameliorated by serotonin synthesis. Furthermore, the administration of probiotic-derived EVs may represent a safe therapeutic strategy for targeting specific sites of inflammation and inflammation-altered microbiomes (Figure 11). Therefore, based on our validation using different models, we propose that the probiotic-derived EVs investigated in this study could be a part of a novel and safe therapeutic strategy to rapidly alleviate diseases without side effects.

## Supplementary Material

LF216EV AD Supplementary materials 240918 revision.docx

## Data Availability

The small RNA-seq and mouse fecal metagenome are available on NCBI BioProject under accession numbers PRJNA1116818 and PRJNA1116804, respectively.

## Abbreviations

AD: Atopic dermatitis
EVs: extracellular vesicles
LF216EV: *Limosilactobacillus fermentum* SLAM 216 derived extracellular vesicles
DNCB: 2,4-dinitrochlorobenzene
