# Supplementary material for: Extracellular vesicles of Limosilactobacillus fermentum SLAM216 ameliorate skin symptoms of atopic dermatitis by regulating gut microbiome on serotonin metabolism
Source: Gut Microbes. 2025 Mar 3;17(1):2474256. doi: 10.1080/19490976.2025.2474256 (PMC11881872; doi:10.1080/19490976.2025.2474256)
Supplement: LF216EV AD Supplementary materials 240918 revision.docx [file KGMI_A_2474256_SM8030.docx]

**Supplementary materials**

**Extracellular vesicles of *Limosilactobacillus fermentum* SLAM216 ameliorate physiological and psychological symptoms of atopic dermatitis by regulating serotonin metabolism through the modulation of gut microbiota**

Hyejin Choi^1#^, Min-Jin Kwak^1#^, Youbin Choi^1^, An Na Kang^1^, Daye Mun^1^, Ju Young Eor^1^, Mi Ri Park^2^, Sangnam Oh^3*^, and Younghoon Kim^1*^

*^1^Department of Agricultural Biotechnology and Research Institute of Agriculture and Life Science, Seoul National University, Seoul 08826, Korea*

*^2^Korea Food Research Institute, Wanju 55365, Korea*

*^3^Department of Functional Food and Biotechnology, Jeonju University, Jeonju 55069, Korea*

^#^These authors contributed equally to the manuscript.

*To whom correspondence should be addressed: osangnam@jj.ac.kr or ykeys2584@snu.ac.kr**
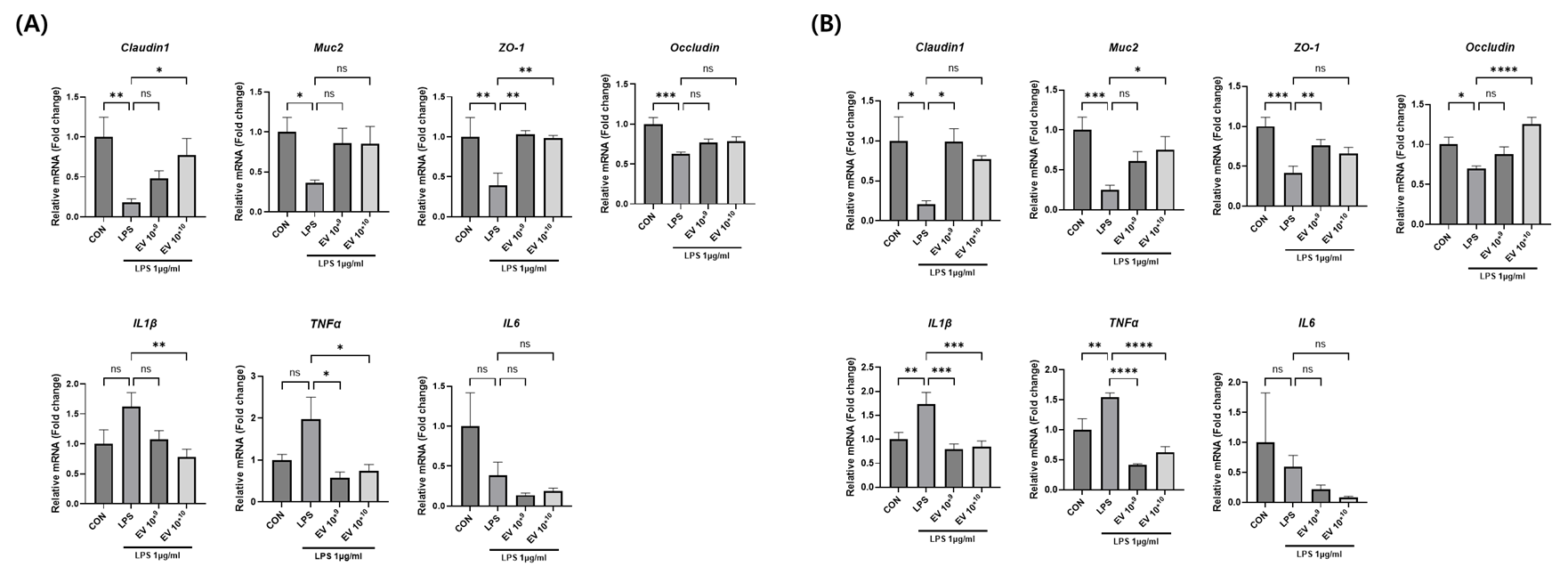
 Supplementary Figure 1. Expression of mRNAs changed by LPS treatment in Caco-2 and HT-29 cells**. (A) qPCR analysis of mRNA expression levels of tight junction-related genes (*Claudin1, Muc2, Zo-1, Occludin*) and inflammatory cytokines (*IL1β, TNFα, IL6*) in Caco-2 cells treated with LPS 1 μg/ml for 24 hours. (B) qPCR analysis of mRNA expression levels of tight junction-related genes (*Claudin1, Muc2, Zo-1, Occludin*) and inflammatory cytokines (*IL1β, TNFα, IL6*) in HT-29 cells treated with LPS 1 μg/ml for 24 hours.**
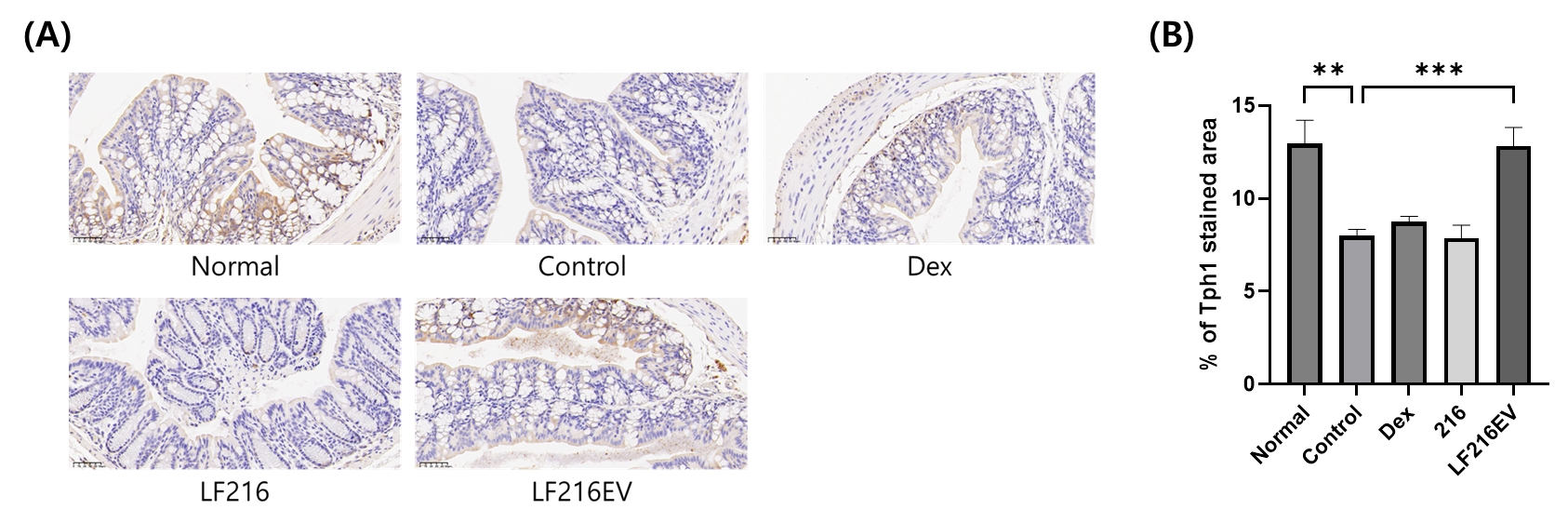
**

**Supplementary figure 2. Expression of Tph1 protein in mouse intestine.** (A) Immunohistochemical staining results of each treatment group. (B) The quantification graph of Tph1 protein staining.

**
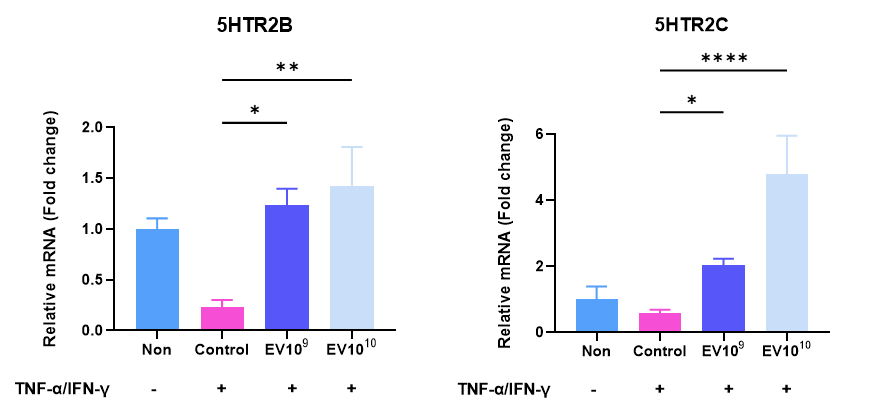
**

**Supplement figure 3.** mRNA expression levels of serotonin receptor genes (5HTR2B, 5HTR2C) in inflammation induced HaCaT cell using qRT-PCR. Data are depicted as mean values ± SEM from samples. * P<0.05, ** P<0.01 and *** P<0.001 vs. the DNCB control.
